# Incisional Small-Bowel Strangulation after a Caesarean Section: A Case Report

**DOI:** 10.3390/medicina60010190

**Published:** 2024-01-22

**Authors:** Agne Plume, Arnoldas Bartusevicius, Saulius Paskauskas, Laura Malakauskiene, Egle Bartuseviciene

**Affiliations:** 1 Department of Obstetrics and Gynaecology, Riga Stradins University, LV-1007 Riga, Latvia; 2 Department of Obstetrics and Gynaecology, Lithuanian University of Health Sciences, 44307 Kaunas, Lithuania; arnoldas.bartusevicius@lsmuni.lt (A.B.); saulius.paskauskas@lsmuni.lt (S.P.); laura.malakauskiene@lsmuni.lt (L.M.); egle.bartuseviciene@lsmuni.lt (E.B.)

**Keywords:** caesarean section, postoperative complication, small-bowel strangulation

## Abstract

*Background and Objectives*: Every surgical procedure has the possible risk of complications, and caesarean sections (CSs) are no exception. As CS rates are increasing worldwide, being familiar with rare but possible complications has become extremely important. *Case report*: We present a case of 25-year-old nulliparous patient who came to our hospital with twin pregnancy for a scheduled induction of labour. An urgent CS was performed due to labour dystocia. On the second postoperative day, the patient started to complain about pain in the epigastrium, but initially showed no signs of bowel obstruction, passing gas, and stools, and could tolerate oral intake. After a thorough examination, an early postoperative complication—small-bowel strangulation at the incision site—was diagnosed. Small bowels protruded in between sutured rectus abdominis muscle causing a strangulation which led to re-laparotomy. During the surgery, there was no necrosis of intestines, bowel resection was not needed, and abdominal wall repair was performed. After re-laparotomy, the patient recovered with no further complications. *Conclusions*: Although there are discussions about CS techniques, most guidelines recommend leaving rectus muscle unsutured. This case demonstrates a complication which most likely could have been avoided if the rectus muscle had not been re-approximated.

## 1. Introduction

The rate of caesarean sections (CSs) is continuously rising, now accounting for 21% of all childbirths globally. Supposedly, this number will increase to nearly a third (29%) of all births by 2030 [1]. Although patients and some medical professionals consider planned CSs an ordinary, safe, and less time-consuming birth method, the increasing number of elective CSs is regarded as an important cause of the rise in CS rates and associated complications [2]. Data from the United States Nationwide Inpatient Sample showed that 76 in 1000 caesarean births were associated with at least 1 of 12 complications (cystotomy, death, anaesthesia complications, placenta accreta, shock, sepsis, renal failure, ventilation support, urinary bladder operation, obstetric wound complications, blood transfusion, and prolonged hospital stays) [3]. While some of CS-associated complications can be diagnosed immediately, it is important to be aware of possible atypical presentations as well. In this report, we present a case of a rare early CS complication—incisional small-bowel strangulation that led to re-laparotomy. Our hypothesis is that this complication most likely could have been avoided.

## 2. Case Presentation

A 25-year-old nulliparous patient with no chronic illnesses or previous surgeries was admitted to the Lithuanian University of Health Sciences Hospital Kaunas Clinics, the Department of Obstetrics and Gynaecology. The patient had a scheduled induction of labour at 38 weeks and 2 days of gestation with a dichorionic–diamniotic twin pregnancy. Labour was induced with vaginal misoprostol followed by oxytocin stimulation. An urgent CS was performed due to the insufficient descent of the foetus and twin A head deflexion.

A caesarean section was performed through Pfannenstiel incision under epidural anaesthesia. After delivering both twins and placentas, the uterus was sutured using single-layer continuous sutures. Visceral peritoneum was sutured separately using continuous suture. Parietal peritoneum was left unsutured, the rectus abdominis muscle was re-approximated and sutured with three interrupted stitches. Transverse fascial incision was closed with slowly absorbable continuous sutures. Subcutaneous tissue was closed with interrupted stitches, skin—with subcuticular running suture. All sutures used were polyglactin 910 (Vicryl) 0 or 1, except for skin which used 2′0.

**Figure 1:**
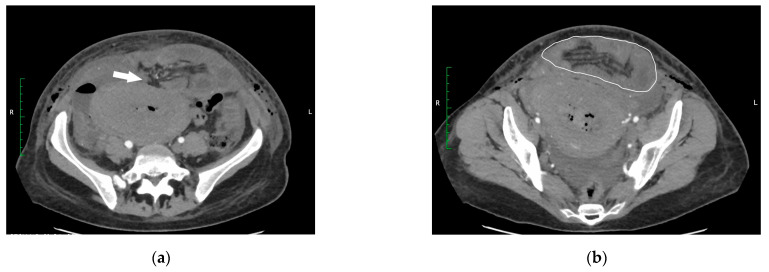
(**a**) CT scan showing an infraumbilical abdominal wall defect (white arrow). (**b**) Eventration of the small bowel (white outline).

The first postoperative day was uneventful. On the second postoperative day, the patient complained about pain in the epigastrium. The abdominal ultrasound scan revealed the free fluid and mild oedema of intestinal walls. Urinary tract injury was suspected since the patient initially showed no signs of bowel obstruction—was tolerating oral intake, passing gas and had bowel movement. A diagnostic ultrasound-guided laparocentesis was performed to confirm the diagnosis. Serohemorrhagic fluid was sent for the creatinine test and culture. The creatinine test and culture were negative, and urinary tract injury was ruled out. On the third postoperative day, the patient complained about diffuse spastic abdominal pain and vomiting. During the examination, the abdomen was diffusely painful with marked tenderness in the epigastrium. Peritoneal signs were negative. The auscultation revealed that hyperactive bowel sounds were present. Abdominal radiography showed signs of small-bowel obstruction. The abdominal-computed tomography (CT) scan revealed an infraumbilical abdominal wall defect approximately 2.5 cm in diameter (Figure 1a) with an eventration containing distal loops of ileum, occupying an area of 12.3 × 6.9 cm (Figure 1b). 

**Figure 2:**
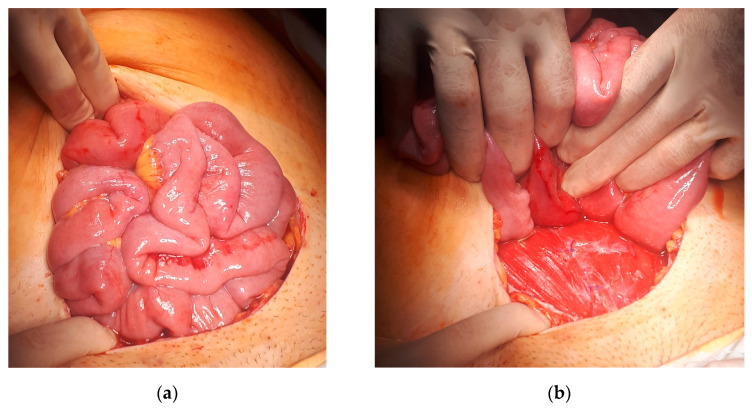
(**a**) Small-bowel loops after the aponeurotic suture was released. (**b**) Small-bowel loops strangulated between two separate muscular stitches with protrusion in between rectal muscles and aponeurosis layer.

During urgent re-laparotomy, after disassembling the aponeurotic suture, small-bowel loops were noted to be strangulated in approximately 2 cm defect which had been left in between two sutures when re-approximating abdominal muscles with interrupted sutures (Figure 2). 

There was no necrosis in the intestines, only small haemorrhages in the mesentery and serosa of approximately 10 cm long segment of the intestine. Around 1500 mL of free fluid was evacuated, and abdominal wall repair was completed. The following postoperative period was uneventful and the patient recovered with no further complications. After a follow-up of 8 weeks after delivery, the patient had no complains and the physical exam was normal. We contacted the woman 2.5 years after the surgery; she had no complaints or any health issues related to the surgery during this period of time.

## 3. Discussion

### 3.1. CS Complications

The history of CS dates back as far as Ancient Roman times and, although it was performed to save the foetus, it had fatal outcomes for the mother. Nowadays, CSs are the most common major obstetrical surgery type with averages ranging from 5% in sub-Saharan Africa to 42.8% in Latin America and the Caribbean. Since 1990, CS rates have risen in all regions around the world [4]. Different methods and modifications for CSs exist, starting with the ones that nowadays are mostly considered a distant past and ending with an extraperitoneal approach. Despite all the discussions about CS techniques, there is no unified consensus on what is the best way to perform this surgery. Standardized surgical steps would allow early and late outcomes to be compared. This would also enable a comparison between obstetricians and institutions in order to provide the best possible care for the parturient [5].

In the long term, CSs are associated with abnormal placentation, uterine rupture in subsequent pregnancy, chronic pain, pelvic adhesions, infertility, and irregular bleeding [6]. Short-term CS maternal complications include pain, endomyometritis, wound infection and/or separation, urinary tract infection, gastrointestinal problems, deep venous thrombosis, and septic thrombophlebitis [7]. 

Re-laparotomy after a CS in the early postoperative period is extremely rare. A cross-sectional study at the Rajshahi Medical College Hospital (Bangladesh) revealed that the re-laparotomy rate in their study was 0.39% (50 cases) with a fatality rate of 18% (9 cases). Abdominal wall repair was needed only in two cases with no fatal outcomes. As the research was performed in a tertiary centre, we have to mention that out of 50 re-laparotomy cases, 42 cases had caesarean deliveries in other hospitals and were referred to the tertiary centre for re-laparotomy [8]. An observational population-based study from the Swedish National Patient Register and the Swedish Medical Birth Register analysed surgical complications after a CS. Long-term complications included bowel obstruction, incisional hernia, and abdominal pain. Short-term complications occurring within 42 days after delivery (bleeding, infection, organ damage, wound dehiscence, bowel obstruction, and others) were assessed as well. This large-scale study included 79 052 primiparas with caesarean deliveries and 402 316 primiparas delivering vaginally—as the control group. Wound dehiscence as a surgical complication after CSs was reported in 0.23%. The bowel obstruction rate was 0.6% (0.09% within 42 days after delivery), but women with comorbidity and previous abdominal surgery were not excluded from the study. Incisional hernia occurred in 1% of cases with a median time of 4.1 years from the CS to the complication diagnosis. Emergency CSs are associated with an increased risk of surgery due to bowel obstruction or hernia complications [9]. Recently, a systematic review showed that incisional hernia rate varied from 0.0 to 5.6% with a follow-up time ranging from 6 months to 10 years. A possible reason for such results is the large number of midline incisions in some developing countries that were included in the research. Based on the included studies, it was not possible to estimate whether the urgency of the CS affected the incisional hernia development [10].

### 3.2. Similar Cases

Our case presents small-bowel strangulation at the incision site. After an extensive literature search, we were able to find three cases describing small-bowel strangulation at the incision site. 

The first case described by C. Van Der Wal et al. was a 38-year-old patient after a third elective CS with tubal ligation. The surgery and initial postoperative period were uneventful, and on the second postoperative day, the patient was discharged home. She was re-admitted 2 days later with abdominal pain and vomiting. In the beginning, she was treated conservatively for paralytic ileus. She recovered and was discharged after having bowel movement. On ninth day, she was referred with acute colicky abdominal pain associated with bilious vomiting. The abdominal X-ray showed distended loops of the small intestine, and a diagnosis of intestinal obstruction was made. During emergency laparotomy, a large defect at the lower part of the rectus muscle (including the peritoneum) was present. Small bowel was densely adherent to the rectus sheath and rectus muscle. Adhesion was released and small bowel loop was reduced in the abdominal cavity, abdominal wall repair was done in layers. The patient recovered with no further complications [11].

A second report by R. Owen and D. Polson presented a case of a 36-year-old primipara after an emergency CS. There were no complications during the surgery and the patient was discharged on the third postoperative day. She was re-admitted 3 days later with severe abdominal pain and vomiting. The CT scan revealed a dilated obstructed ileum. During re-laparotomy, a 30 cm small-bowel segment was present above the rectus muscle and trapped beneath the rectus sheath. Bowel resection and end-to-end anastomosis was performed. The patient recovered and was discharged on the seventh postoperative day [12].

A third report by Z. Marchocki et al. presented a 34 year-old primipara after an elective CS. The patient had no short-term post-operative complications and was discharged on the fourth postoperative day. On the eighth postoperative day, she was re-admitted with a five-hour history of severe lower abdominal pain, nausea, vomiting, and an episode of diarrhea. The initial working diagnosis was endometritis and antibacterial treatment was started. The next day during physical examination, the patient had a tender abdomen with guarding and absent bowel sounds. Inflammatory markers were increased. A CT scan revealed herniation of the small-bowel segment into the anterior abdominal wall defect. During re-laparotomy, the strangulated small bowel was lying above the rectus muscles and trapped beneath the intact rectus sheath. Then, 34 cm of the necrotic bowel was resected and end-to-end anastomosis was performed. Patient had no further complications and was discharged on the ninth postoperative day [13]. 

In all these case reports, parietal peritoneum was left unsutured. There is no information about whether the rectus abdominis muscle was sutured. These case reports address that the non-suturing of the parietal peritoneum might be a cause of small-bowel strangulation at the incision site and encourage a debate around whether a recommendation to not close the peritoneum is appropriate. 

### 3.3. Abdominal Wall Closure

According to the National Institute for Health and Care Excellence guidelines, visceral and parietal peritoneum and rectus muscles should not be sutured, as this reduces the operating time and the need for postoperative analgesia [14]. Despite that, there is some evidence to suggest that the non-closure of the peritoneum after a CS is associated with more adhesion formation [15]. Contrary, a 3-year follow-up of the CORONIS trial proved that there is no difference in any outcomes relating to symptoms associated with pelvic adhesions between peritoneum closure and non-closure groups [16]. It has to be emphasized that in the CORONIS follow-up trial, only involuntary infertility was assessed as a symptom associated with pelvic adhesions. 

Rectus muscle re-approximation and adhesion formation were evaluated in the secondary analysis of the prospective cohort study at the Stanford Medical Centre. Analysis included women who underwent a first repeat caesarean delivery. The closed rectus group included 40 patients and the open rectus group included 125 patients. The results revealed that rectus muscle closure was associated with the formation of less dense adhesions compared to the open rectus group (17.5% vs. 46%) [17]. It is important to highlight that the open rectus group’s patient sample was more than three times higher (125 vs. 40). Several studies have proven that rectus muscle re-approximation increases the need for postoperative analgesia [18,19] and may cause unnecessary pain when the patient starts to move after surgery [20]. There is a study which claims that rectus muscle re-approximation has no effect on the prevention of diastasis recti [21]. Controversially, a more recent study claims that the re-approximation of rectus muscle increases muscle strength and core endurance in the early postoperative period and has a positive effect on decreasing the inter-rectus diastasis [22]. We were able to find one case report which proposes a new modified undermined suture technique for rectus muscle re-approximation during caesarean delivery. After a repeated CS was carried out with the proposed rectus re-approximation technique, the patient claimed that postoperative pain was 2/10. Her abdomen was firmer, flatter, and more stable compared to her previous CS experience where rectus muscle was re-approximated with interrupted stitches [23]. There is a lack of evidence of the technique’s applicability and possible benefits for the patient as it has been reported for only one single case.

Fascial closure remains the critical part of closing the incision. This tissue provides the greatest wound tensile strength during healing. A few randomized trials have evaluated optimal closure techniques for transverse incisions: a continuous non-locking closure with 0 or 1 slowly absorbable braided suture is the common approach, but a monofilament also can be used [24,25]. There have not been any trials evaluating the optimal suture width for fascial closure in transverse incision. In a double-blind, multicentre, randomised controlled trial comparing small bites (5 mm) versus large bites (10 mm) for midline incision fascial closure, significantly fewer patients in the small bites group developed incisional hernia after one year (13% vs. 21%) [26]. 

## 4. Conclusions

The abdominal wall closure after caesarean section is not globally standardised, but most of the guidelines recommend leaving peritoneum and rectus muscle not sutured. This minimises postoperative pain and the need for analgesia as peritoneum and muscles adjust to previous anatomical location on their own. Despite national guidelines, which recommend leaving peritoneum and rectus abdominis muscle unsutured, in this case, parietal peritoneum was left unsutured but rectus abdominis muscle was closed separately from other layers with interrupted stitches. This led to intestinal protrusion and strangulation in between two separate muscular sutures. This case demonstrates a complication, which most likely could have been avoided if rectus muscle had not been re-approximated. It can only be speculated if the closure of parietal peritoneum could have prevented small-bowel protrusion and strangulation in between rectus muscle. With this case, authors would like to emphasise that neither peritoneum nor rectus abdominis muscle should be sutured to reduce operative time and the need for postoperative analgesia.

It is not possible to evaluate or compare the outcomes of a CS as the technique is not standardised. Many surgeons do not follow the guidelines and perform the surgery in a preferable manner. Following guidelines and reporting present complications would lead to optimising CS technique. Global CS standardisation would allow us to compare short-term and long-term outcomes of this most common major surgery in obstetrics and gynaecology, while possibly avoiding known complications associated with surgical techniques.

## Data Availability

The original contributions presented in the study are included in the article, further inquiries can be directed to the corresponding author.
